# Seeking treatment for uncomplicated malaria: experiences from the Kintampo districts of Ghana

**DOI:** 10.1186/s12936-016-1151-7

**Published:** 2016-02-20

**Authors:** Lawrence G. Febir, Kwaku Poku Asante, Samuel Afari-Asiedu, Livesy N. Abokyi, Anthony Kwarteng, Bernhards Ogutu, Margaret Gyapong, Seth Owusu-Agyei

**Affiliations:** Kintampo Health Research Centre, P. O. Box 200, Kintampo, Ghana; Dodowa Health Research Centre, P. O. Box DD1, Dodowa, Ghana; INDEPTH Network, Accra, Ghana

**Keywords:** Uncomplicated malaria, Experiences, Treatment

## Abstract

**Background:**

Malaria accounts for many deaths and illnesses, mostly among young children and pregnant women in sub-Saharan Africa. An integrated approach is recommended to ensure effective malaria control. Socio-cultural factors continue to serve as determinants of malaria health-seeking behaviour. An INDEPTH effectiveness and safety study platform was established to unearth issues around the use of licensed and nationally recommended anti-malarials in real life settings. This study reports on treatment-seeking behaviour for uncomplicated malaria among community members.

**Methods:**

A qualitative study was conducted in the dry and rainy seasons in purposively selected communities in Kintampo north and south districts. This was based on distances to a health facility, ethnicity and availability of medicines at the sale outlets. Twenty-four focus group discussions were conducted among adult men, women care-takers of children less than 5 years and pregnant women. Ten INDEPTH interviews were also conducted among operators of medicine sale outlets and managers of health facilities. Fifty-one illnesses narrative interviews were conducted among adult men, women, women caretakers of children less than 5 years and pregnant women. Transcripts were transferred into Nvivo 8 software for data management and analysis.

**Results:**

The artemisinin-based combinations that were commonly known and used were artesunate–amodiaquine and artemether–lumefantrine. Use of herbal preparation to treat diseases including uncomplicated malaria is rife in the communities. Drug stores were not the main source of artemisinin-based combination sales at time of the study. Monotherapies, pain killers and other medicines were purchased from these shops for malaria treatment. Dizziness, general body weakness and sleepiness were noted among respondents who used artemisinin-based combination therapy (ACT) in the past.

**Conclusion:**

There is no clear cut trajectory for management of uncomplicated malaria in the study area. Different approaches are adopted when treating malaria. There is need for community education to influence behaviour on the management of malaria to achieve real gains from ACT use.

## Background

Malaria is still a major cause of deaths and illnesses; it affects mostly young children and pregnant women with the highest burden in sub-Saharan Africa. It was responsible for an estimated 482,000 deaths among children under 5 years, 3.4 billion people worldwide are at risk of malaria infection, 90 % of all malaria deaths occur in sub Saharan Africa [1]. An integrated approach involving all the currently available tools is recommended. Access to treatment with effective anti-malarials need to be ensured, while also undertaking preventative measures such as targeted vector control [2]. Though some studies have been carried out that described factors influencing treatment-seeking for malaria, far less is known about the relative weight given to these factors in different settings. Sociocultural research on malaria health-seeking behaviour recognizes issues influencing choice of provider for treatment [3, 4]. Other factors such as ethnicity also influences treatment-seeking with certain ethnic groups being much more reliant on traditional self-medication remedies [5, 6]. These sociocultural determinants of malaria health-seeking behaviour are often local and may change with time [7–9].

Product development Phase I, II and III randomized, controlled clinical trials have been well-supported and have established the initial safety and efficacy of such products when delivered under tightly monitored conditions. However, there is very little experience with the effectiveness and safety evaluation of medicines deployed on a large scale within African health systems. An effectiveness and safety platform was established by the INDEPTH network to document the safety and effectiveness of artemisinin-based combination therapy (ACT) in real-life settings. This platform, INDEPTH Effectiveness and Safety Study (INESS) was established with the aim of minimizing the time gap between post-regulatory licensure and adoption of new anti-malarials by providing timely safety and effectiveness data from endemic countries to guide national and global policies and practices.

Qualitative data was collected as part of this INESS initiative with the aim of identifying factors that influence community members’ ability to seek and receive appropriate treatment for malaria in Kintampo in the middle part of Ghana.

## Methods

### Study area

Data was collected from respondents in the Kintampo north and south districts in Ghana, between November 2009 and July 2010. The two districts are located within the forest-savannah transitional ecological zone in the Brong-Ahafo Region, covering an area of 7162 km^2^. Malaria transmission is high with 269 infective bites per person per year with an all year round parasite prevalence of about 50 % among children <5 years of age [10]. The area’s resident population was 134,970 in 2009 [11]. Community members are predominantly farmers. Data were collected to coincide with the dry season (i.e. December 2009 to March 2010), a period that corresponds with low malaria transmission, and collection was repeated during the rainy season (i.e. April 2010 and October 2010), which corresponds to the high malaria transmission season.

The majority of inhabitants in this area initiate treatment for some ailments at home, then continue to the Licensed Chemical Shops and may finally end up in a public health facility if their illness do not resolve [12]. There are two public hospitals, twelve health centres/clinics and thirty community-based health planning and services (CHPS) compounds. These facilities provide health service delivery to urban as well as to the majority of deprived rural poor community members in the study area.

### Data collection methods

Four rural communities located in the south and west of the Kintampo Districts were purposively selected for data collection based on availability/unavailability of health facilities as well as ethnic balance involving the two dominant tribes in the study area.

### Focus group discussions (FGDs)

Twenty-four FGDs were conducted separately, eight among adult men, eight among pregnant women and eight among caretakers of children less than 5 years of age in their respective communities. Compounds having potential discussants were randomly selected using the Kintampo Health and Demographic Surveillance System (KHDSS) database [11]. The selection criteria were: adults between 18 and 80 years who lived in the purposively selected communities. The potential discussants were identified in their various homes through the assistance of community opinion leaders. Respondents of different ages were put together to form a group of discussants. Males and females were put in separate groups for each discussion session. Discussions were held in twi (the widely spoken local dialect) all FGDs were held in either a church or an open space. The discussions were facilitated by a moderator using an interview guide that was made up of pre-determined questions and themes. Other related emerging issues were also discussed. Notes on responses and other non-verbal communications were also taken by a notes-taker. Each session was digitally recorded; this was complemented with the notes taken by the notes-taker during the results write up process. Each discussion session also lasted for about an hour. Interview sessions were brought an end when the moderator had exhausted all questions on the interview guide and other emerging issues. The FGDs assessed the care-seeking behaviour for malaria, knowledge of malaria treatment and malaria treatments received at the health facilities and retail medicines sector. The perception of community members on how malaria treatment works general knowledge and experiences with new anti-malarial drugs and their experiences with ACT, more specifically artesunate–amodiaquine. ACT contraindications were explored; their perceptions of the most effective and the less effective malaria treatment were also explored. A study team member moderated each interview session while the responses were recorded by a second person to aid transcription and analysis. Interview sessions lasted between one and one and half hours and involved discussants who numbered between eight and twelve.

### In-depth interviews (IDIs)

Ten in-depth interviews (IDIs) were conducted by a study team member, among health centre workers (4- 2 in Kintampo North and South Districts respectively) and drug store operators (6- All in Kintampo South District). All the drug store operators and in-charges of health centres in both districts were interviewed. There were no drug stores in study communities in Kintampo North District as at the time of the study). Interviews among drug/chemical store operators were conducted in the premises where the medicines are sold to clients. Interviews were conducted when attendance was very low to avoid interruption by potential clients. Each session was digitally recorded; this was complemented by the notes taken by the notes-taker during the results write- up process. Each interview session lasted for about thirty minutes to an hour. The health centre workers interviews centred on their hours of operation at the various health facilities and the type of services they provide, category of health personnel working in the facility; malaria diagnosis and treatment practices; patients preference for medicines and the reason(s) for their preference; perceptions on efficacy of medicines and their reasons for that perception; the types medicines patients request for, types of anti-malarial drugs in retail outlets and patients knowledge of malaria. In the case of medicine shop operators, interviews centred on the hours of operation at the various medicine shops, the services provided, anti-malarials including ACT available in the shop for sale, the type of ACT stocked and their source of supply; knowledge of malaria, clients medicine preference and the reason(s) for such preference, providers medicine preference and the reasons for this preference.

### Illness narrative interview (INI)

The illness narrative interview was performed using an interview guide on the last episode of malaria, sources and sequence of treatment, preference for any particular type of treatment, perceived side effects and experiences with such treatments, perceptions about efficacious medicines and reasons for preference of such medicines. All persons who were ill with a fever in the past 2 weeks prior to the day of interview were eligible to be interviewed. Caretakers responded on behalf of their under 5 years old children. INIs lasted between 10 and 25 min. All INIs were digitally recorded by a moderator who also made summaries and notes of non-verbal communication. Once the questions on the guide and other emerging issues are exhausted by the moderator, the interview session comes to an end.

A total of fifty-one INIs were conducted, 12 among men, 16 among women, 16 among women caretakers of children less than 5 years of age and 7 among pregnant women. Respondents were individuals in the study area that were part of the KHDSS database and have had an episode of malaria in the 2 weeks preceding the day of the illness narrative interview. Caretakers of children less than 5 years of age responded for their children who were qualified to be interviewed. To select respondents, a list of compounds was randomly generated from the KHDSS database. This was followed by a search through the compounds for any member defined above who qualified for this interview. Those found were screened to ascertain eligibility and to ensure that not more than one category of respondent is selected from a compound. Respondents were interviewed on the sequence and types of treatment sought for that episode of malaria, the content of their consultation with health providers, the type of malaria treatment provided by health care providers, the perceptions of malaria treatment received, their opinions about best malaria medicines and malaria prevention and any malaria related issues in the communities.

### Data management and analyses

Data from FGDs, IDIs and INIs were recorded using a digital recorder, transcribed verbatim, checked for completeness and accuracy; audio-recordings were vetted to match transcripts by the study team leader. The data were coded independently by two persons. To ensure inter-coder agreement and reliability, 10 % of the transcripts were coded and this was followed with a debriefing session to discuss the level of agreement. The level of agreement between the independent coders was always above 90 %. Responses were subsequently grouped and categorized according to the themes under the study objectives and emerging themes as determined by the data using NVIVO version 8 qualitative data management software. Data were thematically analysed to identify themes that best addressed study’s objectives and related emerging themes. Responses that best describe the various themes served as quotes to support the findings. Table 1 gives details on how the data was analysed.

**Table 1:** How data collected in December 2009–October 2010 was analyzed

1	Two moderators coded data independently
2	10 % of data were independently coded and compared to ensure inter coder reliability and an agreement then reached on a coding tree
3	Themes were identified in advance and emerging themes were derived from data
4	Nvivo version 8 software was used for data management
5	Quotations were presented to illustrate themes. All quotes attributable to participants were anonimized
6	Major themes were clearly presented in the findings
7	There were descriptions of diverse cases and discussion of major themes

### Ethical issues

Approval to conduct the INESS study was granted by the ethics committees of Ghana Health Service and the Kintampo Health Research Centre. Discussants and participants in FGDs, IDIs and INIs were taken through an informed consent process; in which they either agreed or disagreed to be part of discussion/interview. Community entry involved explaining the study to chiefs and key community opinion leaders. This was followed by preparatory meetings with community opinion leaders and other stakeholders from these communities to explain details of INESS and the objectives of the qualitative component, what their roles are and would be in the data collection processes.

## Results

### Characteristics of respondents

Table 2 gives a summary of the characteristics of respondents.

**Table 2:** Characteristics of respondents

Data collection method	Age ranges of participants		Education level of participants	
	Men	Women	Men	Women
Focus Group discussion	25–80	18–45	From no education to tertiary	From no education to senior high school
INDEPTH Interview	35–52	53–56	From middle School leaving certificate to senior high school	From middle school leaving certificate to Midwifery
Illness narrative interview	36–60	18–55	From no education- to general certificate of Education (ordinary level)	From no education to senior high school

### Community knowledge of ACT and availability of ACT at the community level

The artemisinin-based combinations that were commonly known and used by community members are artesunate–amodiaquine and artemether–lumefantrine. Artesunate–amodiaquine was commonly referred to as the “Yellow and white” tablets by respondents:

when I took that white and yellow, I only felt weak. That is the only thing I saw, apart from that, I did not experience any other side effect (FGD: Man)

He gave me drugs; one is white and the other is yellow. He also gave me blood tonic and para. (INI: Woman)

In some of the communities accessing ACT was a challenge. ACT was not available for community members to buy in time of need.

The white and yellow isn’t available at the drug store (FGD, pregnant woman)

Some community members mentioned only “amodiaquine” when they actually meant *artesunate*–*amodiaquine*. Artesunate–amodiaquine, which is the first-line drug for the treatment of uncomplicated malaria is the ACT usually dispensed at the health facilities. Artemether–lumefantrine is dispensed by some health facilities in the study area.

As said earlier, amodiaquine is very good for the people of Amoma [referring to artesunate-amodiaquine] (FGD, Man)

The medicine sale outlets (Drug stores) do not normally dispense ACT. Community members therefore purchase monotherapies, pain killers and other drugs from them to treat malaria.

That is what I told you earlier that we do not sell drugs like artesunate–amodiaquine but we sell drugs like Quick Action [Caffein, Ephederine HCL, paracetamol] and efpac [acetylsalicylic acid, caffeine, paracetamol] which are always advertised on radio so if the person comes and we have to give him Efpac or Quick action, we tell him to take it three times a day (IDI, drug shop operator)

For malaria medicines we have malafan [sulfadoxine pyrimethamine, Amodiaquine syrup; first we have the tablet but now we do not get it that much and Kinaquin 442 [Chloroquine]. (IDI, Drug shop operator)

### Experience with ACT

Respondents reported several experiences when they used ACT. Respondents generally held that, ACT make them weak and as such make them feel sleepy a lot and that one needs a long rest when taking ACT.

The drug causes you to sleep so when you take it you need to rest for about two to three hours else you become very weak (INI, woman)

Also respondents at INI sessions commonly mentioned that ACT can cause dizziness if a patient takes it without eating

Personally I feel dizzy when I take artesunate-amodiaquine without eating (INI: Man)

Others also mentioned that ACT cause them to vomit.

I came home just around this time. He asked me to take some of the drug so I took it. I also took some around one o’clock in the afternoon; he gave me three and asked me to try and take all in three days. So I took two in the morning and two in the afternoon; whilst taking it I felt the scent of the drug in my nose but I tried to swallow it. After taking it I felt like vomiting but I tried as much as possible to control it but I couldn’t so I had to vomit; that day I vomited a lot. (INI; pregnant woman)

Again, respondents mentioned that for ACT to work well and to prevent body weakness the patient must eat before taking it, apart from this they also mentioned the need to take the medicines as prescribed. Furthermore, respondents mentioned that ACT cause fast heart beat which will lead to some form of worry as mentioned below by respondent

…So I took two and half for two times, the moment you take the drug you feel dizzy. In about thirty minutes I felt sleepy, so I slept meaning the illness itself was cured. But with the sleep, no break, so my husband even asked whether I’ve taken some alcohol. (INI, Woman)

Respondents associated cure during a malaria episode with the following experiences during ACT treatment: “good sleep”, “urinate very well”, change in urine colour, excessive sweating, “yellow vomit”, eating “very well”, and reduction of a “child’s temperature and chills”.

When you sleep three hours after taking the drug you will sweat and when you urinate you will see the difference. Thus the colour of your urine will change. (FGD: Man)

So after taking the drug, you realize that your temperature comes down; and with the weakness, you not even able to go out to urinate. So, having taken the drug and when it begins to work, you realize you’ve gained some strength and you‘re able to move about. (FGD: Pregnant woman)

### Malaria health-seeking behaviour: the role of ACT

Study participants do not adopt a single treatment-seeking pattern for uncomplicated malaria. Some participants visited health facilities immediately they felt unwell. Those diagnosed for malaria in the health facilities were readily treated with an ACT as the only drug used for malaria treatment. Other community members visited drug stores to purchase any drug they deemed appropriate for the disease suspected or mentioned their health condition to the vendor who decides on which drug (among those listed in Table 3) is most appropriate to be dispensed to cure the illness. Other community members initiated treatment with some left over drugs or start with some herbal preparations using herbs listed in Table 4. There were however, some respondents who used ACT alongside herbal preparations, whiles others used ACT for some days and switch over to any other drug or herbal preparation.

**Table 3:** List of medicines sold to patients in the study area who present with fever/malaria at chemical stores: December 2009–October 2010

Type of anti-malarial monotherapy	Other medicines
Malafan (Sulfadoxine pyrimethamine)	Paracetamol
Kinaquin 442 (Chloroquine)	Oral Rehydration Salt
Chloroquine	Glucose
Camoquine (Amodiaquine)	Cafenol (Acetaminophen, Caffeine)
Amodiaquine syrup	Vitamin B complex
Amodiaquine suspension	Quick Action (Caffein Ephedrine HCL, paracetamol)

**Table 4:** Table showing herbal plants used to treat uncomplicated malaria in the study area: December 2009–October 2010

Local name of plant mentioned by respondents	English name of plant	Scientific name
“Duagyene-wuraa”	“Neam tree Leaves”	*Azadirachta indica*
“Kashedua wuraa”	“Acacia tree Leaves”	*Acacia glauca*
“Acheamponwuraa”	“Siam weed”	*Chromolaena odorata*
“Amango-wuraa”	“Mango leaves”	*Magnifera indica*
“Brofrewuraa-a-awo”	“Dried pawpaw leaves”	*Carica papaya*
“Teek-wuraa”	“Teak leaves”	*Tectona grandis*
Ankaa-twadee-wuraa	“Lime/Lemon leaves”	*Citrus vulgaris*

I agree with him. When you treat it traditionally and it still persists then opt for orthodox treatment in the health facility. (FGD, Man)

I think that even if the herbs will help, one has to go to the hospital first to get treatment, so that upon returning, the herbs can be used to supplement so that it will help. So the hospital is the best way to help ourselves in treating malaria (FGD, Woman)

we only pushed ground ginger into her anus but it didn’t work so I bought drugs at the drug shop. it did not work either so I took her to the hospital. (IDI, caretaker of a child less than five years)

You send the patient to hospital and after that you also cut some herbs like mango leaves, lemon and the “Timforgor” [the name of a herb] that she mentioned and the lemon leaves are mixed together (FGD, Woman)

Community members who used herbal preparations used parts of plants (roots, barks and stems and leaves) as itemized in Table 4.

### Category of people who must not take ACT

The following categories of people were cited by a minority of community members as the group who could not take ACT: pregnant women, children less than 12 years of age, diabetic patients, anemic persons and individuals with heart problems. Community members believed that ACT works on “blood” and therefore likely to affect foetus and children who are perceived to have very little blood. The following quotation show this position

Pregnant women should not take it because from what people say the drug is powerful and melts blood so I think it is not good for them. (FGD: Man)

I said an anaemic; because the drug is powerful and works with blood so such a person should not take the drug. (FGD: Man)

These perceptions were however held by the minority of discussants upon subsequent probing.

### Reasons for self-treatment, care-seeking at health facilities and chemical shops for uncomplicated malaria in the communities

Generally, participants cited different reasons why they self-treat ailments such as malaria. Key among them were lack of money to either purchase medicines at the chemical shops or pay for cost at the health facilities, unavailability of means of transport in their communities to send them to a health facility and poor roads. Self-treatment was practiced to complement treatment that they receive at health facilities or when they perceive that treatment at the health facility failed. Another reason was patronizing the purchase of medicines of drug peddlers during their home visits to convince them of the potency of their medicines. Some quotes below illustrate some reasons cited for self- treatment:

If you realized you have malaria. We have transport problems to help us get to the health facility. You pluck some leaves, boil and take them (IDI, caretaker of a child less than five years)

Supposing you get malaria and have to go to the hospital, you’ll be required to pay; so if you don’t have money you give up and look for some herbs (FGD, Man)

My two year old child has been affected with malaria, and I’ve tried several times but did not work so I’m thinking of preparing herbs, as a support to see if that can work. So I believe that one can help (FGD caretaker of a child less than five years).

The following reasons were however cited for visiting health facilities: services rendered and the availability of laboratory services in the health facilities, the use of health insurance as a mode of financing services delivered at the health facilities, proximity to health facilities and opportunity to visit and report the inability of a medicine provided at the facility to treat their disease condition, lack of knowledge of the cause of a disease condition they have been affected with as well as not getting cured after self-medication or seeking treatment at the drug store. They also cited the devastating effect of malaria and visits to health facilities is in response to calls to seek treatment at health facilities for all ailments, including malaria as some of the reasons. The quotes below illustrate some of the reasons cited for visiting health facilities:

At the clinic, they have some machine. They will first take samples of your blood is to examine whether malaria parasites are in or not. So if it is found out that the parasites are in, you are given malaria treatment (FGD, man)

When the illness comes, I buy drugs but when it becomes very serious, I have to send the person to the health facility. (FGD, man)

The reasons cited for patronizing medicine in retail outlets (i.e. drug stores) included: distance to health facilities, if drugs given at the health facilities are perceived not to be working well, as well as drug stores being immediate and accessible for solutions to health problems and medical advice

The health facility is very far from here as a result after the day’s work or when we wake up and feel body pains all over, we get some pain killer from the drug store before we look for other source of treatment (FGD, Man)

If let us say I send the child to the clinic today being Friday, I’ll wait for a week to see if the child’s condition does not improve, then I’ll buy drugs at the store. I will not send him/her to the clinic again (FGD, caretaker of a child less than five years)

### Malaria diagnosis and treatment practices

Malaria diagnosis was based on clinical symptoms presented at health centres and drugs stores. The excerpts below support this finding.

…when you look at the person and the way he is standing, thus if the person says I am feeling cold, dizzy then you realized that it is fever (IDI, Drug store operator).

We don’t have a laboratory so we look at symptoms to prescribe but when we give them the treatment too they become alright (IDI, an in charge of Health Centre).

This notwithstanding health centre workers and drug store operators considerably adhere to the malaria treatment guideline for children under 5 years of age and pregnant women as revealed in the responses below.

For the under-fives, if we check and he is below the age that he cannot take the Amodiaquine and he is feverish we give para [paracetamol] and advice that they are taken to the hospital (IDI, a drug shop operator).

We do not give any pregnant woman drugs over here. At our workshops we are thought that when someone is pregnant it is only the doctor who can prescribe medicine for her so we do not sell malaria and other drugs to pregnant women (IDI, a drug shop operator).

### Category of staff, hours of operation and type of services provided in health centres and chemical stores

The categories of staff at Health Centers are mainly enrolled nurses and Physician Assistants who are in-charge of consultation and health service delivery. They are supported by Community Health Officers who are in charge of history taking and medicine dispensary. Chemical stores are operated by chemical sellers who have received some basic form of training in medicine dispensary from the pharmacy council of Ghana. This is what a respondent had to say about the category of staff who work at the health center.

I am a physician assistant but you know we have community health officers over there (she points to a section of the health facility). If any FP [Family planning] patient comes, they go to their [community health officer] office and I will be working at the consulting room (IDI, In-Charge, Health Center).

With regards to the hours of operation, whilst health centers operate from 8 a.m. to 5 p.m. on working days, drug shop operators work from 6 a.m. to 8 p.m. a daily on both week days and weekends. However, it is interesting to note that both health centers and drug shops operate as and when they are called upon to attend to patients/clients as illustrated in the quote below.

I work from 8 am to 5 pm on week days; after that when any case comes they come and call me sometimes on weekends. So I’ll work for 24 h (IDI, In-Charge, Health Center).

Health services provided by health centers are mainly diagnosis and treatment of uncomplicated malaria. Other services include the provision of reproductive and child health care such as family planning services, antenatal and post-natal care. Like the health centers, chemical sellers provide treatment for basic ailments based on clinical symptoms but refer children and other clients whose condition they perceive as “higher than” them to the health centers.

For the under-fives if we check and he is below the age that he cannot take the Amodiaquine and he is still feverish we give paracetamol and advice that they are taken to the hospital (IDI, drug shop operator).

Table 5 gives a summary of category of staff, hours of operation and the type of services provided in health centers and chemical stores.

**Table 5:** Category of staff, hours of operation and the type of services provided in health centres and chemical stores in December 2009–October 2010

Category of staff	Hours of operation	Type of services provided
*Health Centre*		
Staff in-Charge of Health Centre (Enrolled nurse or a Physician Assistant)^a^	8 a.m.–5 p.m. during week days (official hours). A patient could call in at any time during the night or over the weekend	(1) In-charge of the day-to-day work at the health centre (2) Serve as consulting nurse in most medical cases that are reported at the health Centre including diagnosing and treatment of uncomplicated malaria (3) Refer patients to referral hospitals for further treatment if the patient’s condition is beyond their capacity
Staff in-charge of history and records (community Health)	8 a.m.–5 p.m. during week days (official hours). A patient could call in at any time during the night or over the weekend and could be called to assist consulting nurse	In charge of taking medical history and keeping of records of facility
Staff in-charge of consulting (enrolled nurse or a Physician Assistant)	8 a.m.–5 p.m. during week days (official hours). A patient could call in at any time during the night or over the weekend	Serve as consulting nurse in most medical cases that are reported at the health centre including diagnosing and treatment of uncomplicated malaria
Staff in-charge of dispensary (Community Health Nurse)	8 a.m.–5 p.m. during week days (official hours). A patient could call in at any time during the night or over the weekend and could be called to assist consulting nurse	In charge of dispensing of drugs to patients who call at the health centre
*Chemical store*		
Chemical store owner^a^	8 a.m.–5 p.m. during week days. A patient could call in at the drug store at any time during the night or over the weekend	(1) Diagnostics (2) Sale of medicines including anti-malarials (3) Counsel and advice clients to seek treatment at the health centre
Chemical store assistant	Work from 8 a.m.–5 p.m. during week days. A patient could call in at the drug store at any time during the night or over the weekend. The assistant acts in the absence of the drug shop owner	(1) Diagnostics (2) Sale of medicines including anti-malarials (3) Counsel and advice clients to seek treatment at the health centre

^a^Main respondent in the IDIs

## Discussion

There have been several studies on the knowledge of ACT among communities and health workers in sub-Saharan Africa [12–16] and their use can be successfully integrated into the malaria home management programme [13]. However a study in Nigeria [14] revealed that a significant number of community members have no knowledge of ACT, though they could have been treated with ACT. This study adequately demonstrates some level of knowledge of ACT among community members that were involved in this study. The challenge about knowledge is the ability of discussants/respondents to mention the name of the actual ACT that they mean. This call for the need for national malaria programme managers to come out with short names or abbreviated versions of names of ACT. Community members should be adequately educated on this abbreviated versions or short names to enhance its usage. Disposition related to medicine use vary among communities. They are either negative or positive. Whilst a study in Malawi recorded positive disposition about use of artemether–lumefantrine [15], this study recorded some negative experiences by community members who used Ghana’s first-line malaria treatment, artesunate–amodiaquine. Some of the negative experiences were dizziness, drowsiness, “body weakness”. These experiences could create perceptions that may have negative consequence for the successful implementation of health programmes, as happened in Nigeria during a polio immunization campaign [16], if not properly managed.

Experiences of patients and community level interpretation of the use of medication has the tendency to foster continuous use of such medicines or rejection. In a study to assess the barriers to malaria mass drug administration [17], it was revealed that fears of perceived side effects such as dizziness, stomach ache and the quantity of medicines that needed to be taken contributed to poor adherence. Women who intended to get pregnant refused and the state of their pregnancy also contributed to the refusals. This study recorded similar findings. Respondents indicated that they feel dizzy when they take ACT. A minority group of respondents cited specific group of people such as pregnant women children and diabetics as people who should not take ACT. This position was not corroborated by Dial et al. [17]. Though this perception was held by a minority group, it is likely that women and children who are at risk may be discouraged from taking ACT. This could lead to poor treatment outcomes. National malaria control programme managers should strengthen education on adherence to curtail this misconception. Another study [18] on adherence and uptake of ACTS in northern Ghana also did not indicate the caliber of people that must not take ACTs. In light of the effects these may have on compliance, experiences in the malaria control efforts studies have come up with some suggestions. A study in Tanzania [19] suggested incorporating local beliefs and practices surrounding use of anti-malarials into programmatic goals to significantly improve uptake malaria control interventions. In Malawi [15], caregivers deliberately altered dosing regimens of artemether–lumefantrine and dihydoartemisinin–piperaquine depending on whether they perceived the medication to be strong or weak. The study by Eving et al. [15] suggested further optimization of anti-malarial adherence among children, with the development of anti-malarials with pharmacological properties that allow user-friendly administration with easy and simplified dosing schedules.

The use of herbal preparations in treatment of diseases is a well-documented phenomenon in many parts of Africa [20–24]. Ethno pharmacological studies on the use of herbal remedies for malaria in Ghana [25] concluded that the use of herbal remedies to treat malaria was a common practice. This study also documents the continual use of herbal preparations in treating malaria. Many unexpected health problems could arise if this not checked and properly managed. Sometimes, doses are not too clear to the end user, chemical composition and toxicity effects are also not clearly documented. These and other contributory factors could make end users vulnerable to other health problems that may lead to fatalities including deaths.

Treatment practices including self-medication with anti-malarials has been widely reported in many parts of Africa [26–31], reasons cited were health facilities challenges such as shortages of medicines, long waiting time, perceived quality of services, location of facilities and distance from homes, inability to pay for health care costs, and lack of freedom to choose medicines of their choice [27, 32]. Drug shops still remain one of the significant sources of anti-malarials distributed to communities [26]; though the main source of anti-malarials, the knowledge of those in-charge of the drug shops was not satisfactory when it comes to malaria case management and other diseases as previously documented [26]

Also, the findings on malaria diagnosis and treatment practices corroborates the fact that, the lack of laboratories and malaria test kits in lower health facilities are the major setback in the diagnosis and treatment of malaria in Ghana. As regards the WHO malaria treatment guidelines [33] adopted by Ghana suspected malaria cases are to be tested and confirmed before malaria drugs are prescribed. There are efforts by the Ghana Health Services to supply malaria test kits to health centres but the supply has been erratic

The categories of health staff in rural health facilities play a major role in the health delivery process. The absence of qualified staff serves as a big disincentive to rural and deprived communities. Their continuous presence in the health facilities and how easily they are accessible contributes immensely to the timely provision of cure for ailments including uncomplicated malaria. The finding on the category of staff in health facilities in this study is in agreement with findings from a systematic review on motivation and retention of health staff in rural communities in developing countries [34]. The review emphasized that, health worker retention is therefore critical for health system performance and efforts must be put in place to ensure their motivation [34].

## Conclusion

There is no clear-cut trajectory for management of uncomplicated malaria in the study area. Different approaches are adopted when treating malaria. Many factors determine how community members seek health when they are unwell. These include drug availability, sociocultural behaviour and cost. The use of herbal preparations to treat many ailments including uncomplicated malaria is very common. There is need for community education to influence behaviour on the management of malaria to achieve real gains from ACT use.
